# Oral Health on Sal, Cape Verde: A Population-Based, Cross-Sectional Study

**DOI:** 10.1016/j.identj.2026.109677

**Published:** 2026-06-11

**Authors:** Catherine M.C. Volgenant, Hans J.J. de Soet, Trynke Hoekstra, Denise Duijster

**Affiliations:** a Department of Cariology, Academic Centre for Dentistry Amsterdam (ACTA), University of Amsterdam and Vrije Universiteit, Amsterdam, The Netherlands; b Department of Preventive Dentistry, Academic Centre for Dentistry Amsterdam (ACTA), University of Amsterdam and Vrije Universiteit, Amsterdam, The Netherlands; c Department of Health Sciences and Amsterdam Public Health research institute, Vrije Universiteit, Amsterdam, The Netherlands; d Department of Oral Public Health, Academic Centre for Dentistry Amsterdam (ACTA), University of Amsterdam and Vrije Universiteit, Amsterdam, The Netherlands

**Keywords:** Public health, Dental Health Surveys, Epidemiology, Dental care for children

## Abstract

**Objectives:**

Sal is one of the ten islands of Cape Verde, a lower-middle-income country in Africa. Local reports suggest poor oral health among residents, but no scientific data exists. This study aimed to assess oral health status and oral health-related quality of life (OHRQoL) of Sal’s dentulous inhabitants, identify risk factors associated with oral health, and explore correlations between oral health and OHRQoL in adults.

**Materials and methods:**

A population-based cross-sectional study was conducted among dentulous Sal residents. Final-year dental students from the Academic Centre for Dentistry Amsterdam (ACTA), Faculty of Dentistry in Amsterdam, performed duplicate oral health examinations. Outcomes included caries experience (dmft/DMFT), consequences of untreated caries (pufa/PUFA), and periodontal health (DPSI; in adults only). Data on brushing frequency, toothbrush ownership, toothpaste availability, dental visits, dental insurance, and tobacco use (adults only) were collected. Adults completed the OHIP-14 questionnaire (OHRQoL). Descriptive statistics summarized characteristics; multivariable regressions assessed associations.

**Results:**

A total of 1,371 dentulous residents participated (609 children aged 1-17 years; 762 adults aged 18-89 years). Caries prevalence was 87.5% and pufa/PUFA 52.2%. Mean dmft/DMFT was 3.7 ± 3.2 (children) and 7.5 ± 6.2 (adults). 63.1% had DPSI maximum-scores ≥3+ (periodontal disease). Toothbrush ownership was high (>96%), access to toothpaste (<90%) and dental insurance was limited (children 63%; adults 46%). Dental insurance was associated with lower dmft/DMFT (*p* = .019) and lower pufa/PUFA (*p* = .005). Higher DMFT, PUFA, and severe periodontal disease was associated with poorer OHRQoL.

**Conclusions and clinical relevance:**

Oral health among Sal’s residents is poor, with a high disease burden and limited access to care. Improving preventive strategies and access to dental services is important to reduce disease prevalence and enhance quality of life.

## Introduction

Oral health is a fundamental part of general health and well-being and contributes significantly to our quality of life.^1^ Globally, oral diseases affect nearly 3.5 billion people, with untreated dental caries in permanent teeth being the most prevalent condition.^2^ These conditions are largely preventable, yet persist due to a combination of factors, including poor access to care, low health literacy, and the increasing availability of sugar-rich diets.^3^^,^^4^ Despite its importance, oral health remains a neglected topic of public health, particularly in low- and middle-income countries (LMICs), where access to and availability of preventive and curative dental services are often limited.^5^, ^6^, ^7^

Cape Verde is an archipelago located off the northwest coast of Africa, off the northwest coast of Africa and approximately 500 kilometres west of Senegal. It is classified as a lower-middle-income country (LMIC) by the World Bank.^8^ The island of Sal, one of its 9 inhabited islands, has undergone rapid economic transformation in recent years, driven largely by tourism.^9^^,^^10^ This growth has led to changes in infrastructure, employment, and food availability. This tourism-driven transformation is not unique to Sal, as tourism in Cabo Verde is also concentrated on specific other islands, particularly Boa Vista. Hence, the intensity of tourism, infrastructure, economic activity, and food environments differs between islands. Therefore, findings from Sal may be relevant to other tourism-oriented parts of Cabo Verde, but should not be assumed to be directly generalisable to all islands.

Although the availability of fresh produce on Sal has increased in recent years, the economic development and reliance on imported processed foods have increased local sugar consumption. Similar trends have been observed in other LMICs undergoing economic transition, where dietary shifts have contributed to increased rates of dental caries and other chronic diseases like obesity, with common risk factors such as diet, hygiene, and stress.^11^, ^12^, ^13^

A previous qualitative study on Sal has similarly highlighted concerns about a high prevalence of untreated dental caries.^14^ However, empirical data to support these claims are lacking. In many LMICs, oral health surveillance is underdeveloped, and available national data are often outdated or incomplete. This lack of surveillance data regarding the oral disease prevalence hinders the development of effective, evidence-based policies and programs to improve local oral health.^15^^,^^13^ Improving oral health can also have a wider economic impact by reducing healthcare costs and minimizing productivity losses.^16^

Oral Health-Related Quality of Life (OHRQoL) reflects the impact of oral health on daily life and is commonly assessed with the Oral Health Impact Profile (OHIP-14) questionnaire. These insights into how oral health affects individuals’ QoL subjective assessments are particularly important in settings where clinical services are limited, as they highlight the experiences of those affected by oral diseases.^17^ Combining both clinical and self-reported data allows for a better understanding of oral health needs and priorities in a specific setting.^18^

Understanding the local oral health status and related risk factors of oral health in a specific setting is essential for developing interventions aimed at preventing and controlling oral diseases.^19^^,^^20^ This study therefore aims to 1) assess the oral health status (caries and its clinical consequences, together with inflammation of the periodontal tissues) and OHRQoL of dentulous residents on the island of Sal, Cape Verde, 2) to identify oral health related risk factors associated with the oral health status and 3) to explore the correlation between oral health and self-reported quality of life among adult dentulous participants. To our knowledge, this is the first large-scale, population-based study of oral health conducted in Cape Verde.

## Materials and methods

### Design and study population

A population-based cross-sectional study was performed on Sal, Cape Verde. Data collection took place between October 2016 and July 2017. All dentulous residents of Sal were invited to participate in the study, with the exception of individuals who were edentulous. No age restrictions or other exclusion criteria were applied for participation in this study, in order to obtain a broad representation of the population at Sal.

To ensure population diversity, recruitment was conducted across a broad range of community settings frequented by local residents, including 3 different schools, the local prison, a community center, a Red Cross post, 2 psychosocial support centers, a surf hub, a health center, and the public library. Recruitment was widely advertised through multiple channels, including flyers, radio announcements, word-of-mouth, visits to schools, community centers, nursing homes, beaches, urban and rural areas, and local businesses such as surf schools. Oral examinations were conducted at these diverse locations.

According to the 2016 statistical yearbook published by the National Institute of Statistics of Cape Verde, the island of Sal had in that year a population of 35,268 inhabitants.^21^ No formal power analysis was conducted prior to data collection; instead, the study aimed to include as many participants as possible to maximize representativeness.

All participants (or their parents/guardians in the case of minors) provided written informed consent. Verbal assent was obtained from all participating children prior to data collection, in accordance with ethical guidelines for research involving minors. As a token of appreciation, participants received a toothbrush and a tube of toothpaste. Individuals experiencing dental pain were after participation referred to a local dentist, mainly for extractions.

The local municipality of Sal granted permission to conduct the study. Due to the absence of a national ethics review committee in Cape Verde, the study protocol was reviewed and approved by the Medical Ethics Review Committee (METC) of the Vrije Universiteit Amsterdam, the Netherlands (reference number: 2016.449, appendix A). The study was reported in accordance with the GROESBE (Guidelines for Reporting Oral Epidemiologic Studies to Inform Burden Estimation) guidelines.^22^

### Data collection: oral health status

Oral examinations were conducted by seven final-year dental students from the Academic Centre for Dentistry Amsterdam (ACTA, the Netherlands). The students travelled to Sal in supervised groups as part of an international internship and performed the examinations during their stay on the island. All students were trained and calibrated before data collection and worked under supervision of experienced dental dentists from ACTA and in collaboration with the local partners. All clinical oral examinations were performed in non-clinical, community-based settings. Consequently, a portable setup was employed to ensure consistent and adequate examination conditions across all locations. This setup consisted of a collapsible chair, a headlamp for illumination, and reusable examination instruments (mouth mirrors and Williams periodontal probes). Infection control procedures were based on ACTA’s standard infection prevention protocols, adapted to the local clinical fieldwork setting. These included the use of gloves, masks, disposable materials, and disinfection and sterilization protocols for the reusable instruments.

Both primary and permanent dentitions were examined for dental caries and clinical consequences of untreated caries. Periodontal screening was performed in adults only. Dental caries was assessed at the surface level using the decayed, missing, and filled surfaces (dmft/DMFT) indices, of which the ‘d/D’ component (dental caries) was recorded according to WHO criteria, which identify lesions only when cavitation into dentine is visible.^23^ This corresponds approximately to ICDAS codes 3 or 4 to 6, thereby excluding early enamel lesions from the analysis. With the dft/DFT values, the Restorative Care Index (RCI) was calculated: the number of filled teeth (FT) divided by the sum of decayed teeth (DT) and filled teeth (DT), expressed as a percentage (FT/(DT+FT)*100).The RCI represents the proportion of decayed teeth that have been treated with fillings, in which a low RCI represents a high proportion of untreated caries.

The pufa/PUFA index was used to document the clinical consequences of untreated dental caries, specifically noting the presence of pulpal involvement (p/P), ulceration (u/U), fistula (f/F), and abscess (a/A).^24^

Periodontal health was evaluated in adults only, using the Dutch Periodontal Screening Index (DPSI). The DPSI is an ordinal scoring system ranging from 0 to 4, used to assess periodontal health based on probing depth, bleeding on probing, and the presence of calculus, with higher scores indicating more severe periodontal conditions. The index dictates that the mouth is divided into sextants, excluding third molars, and the highest score per sextant was recorded based on probing depth, bleeding on probing, and the presence of calculus.^25^ In this study, the highest score observed in any sextant was registered as the participant’s overall DPSI score. The DPSI scores were dichotomized to indicate the presence or absence of periodontitis, with 'periodontitis present' defined as having a DPSI score of 3+ or 4 in at least one sextant.

### Data collection: reliability of oral health measurements

Prior to data collection, all examiners underwent theoretical and clinical calibration training by an experienced dentist for scoring dmft/DMFT, pufa/PUFA and DPSI to standardize diagnostic procedures and minimize inter-examiner variability. Each participant was examined independently by 2 students at the same moment, so all clinical data was collected twice to ensure data reliability and to reduce observer bias. Inter-examiner reliability was assessed using Cohen’s Kappa coefficient for all 3 indices and ranged for dmft/DMFT from 0.70 to 1.0, for pufa/PUFA from 0.97 to 1.0 and for DPSI from 0.8 to 1.0. Intra-examiner reliability was not measured due to the nature of the study.

To assess the reliability of the clinical indices that were measured twice, intraclass correlation coefficients (ICCs) were calculated for the dmft/DMFT and pufa/PUFA scores. The ICC for dmft/DMFT was 0.99 (95% CI: 0.99–0.99), indicating excellent reliability between the 2 sets of scores. The ICC of the 2 sets of pufa/PUFA scores was 0.98 (95% CI: 0.98-0.98), also indicating excellent reliability. A 2-way mixed-effects model with absolute agreement was used, to assess consistency between the repeated measures. For periodontal status, inter-rater agreement on DPSI scores (ordinal data) was evaluated using Cohen’s Kappa coefficient. The resulting Kappa was 0.97 (*p* = .006), reflecting excellent agreement on periodontal status classification.

### Data collection: factors related to oral health

In addition to the clinical examinations, questionnaires were used to collect self-reported data like oral hygiene practices and demographics (age in years and gender (male/female)). Children received the same questionnaire, excluding the OHIP-14 items, which were administered only to participants aged 18 years and older; for children up to 12 years of age, parents or guardians were asked to assist in completing the questionnaire. In Cabo Verde, Cape Verdean Creole is the main language used in daily life, while Portuguese is the official language and the language in education and administration. The questionnaires were therefore administered in Portuguese. When needed, questionnaire items were clarified orally by Creole-speaking members of the fieldwork team (local collaborators) who are familiar with the linguistic and cultural context of Sal. Minor wording adjustments were made to improve comprehensibility, without changing the conceptual meaning of the questions. No formal linguistic validation or pilot testing of the questionnaire was conducted in the Sal population, which is acknowledged as a limitation.

Oral hygiene practices were assessed by inquiring about the weekly frequency of toothbrushing (open response), whether the participant (yes/no) owned a toothbrush, whether toothpaste was available in the household (yes/no) and whether the participant used toothpaste when brushing (never / sometimes / always). Professional oral health care utilization was evaluated by asking whether the participant had ever visited a dentist (yes/no) and if so, how many years ago the last visit occurred (open response). Additionally, participants were asked about the presence of general insurance coverage (yes/no) and dental insurance (yes/no). Other questions included current smoking (yes/no) for adults and whether the participant was currently experiencing oral pain.

### Data collection: oral health-related quality of life (OHRQoL)

To assess the broader impact of oral health on daily functioning and well-being, adult participants completed the Oral Health Impact Profile-14 (OHIP-14), a validated instrument that measures oral health-related quality of life across seven domains: functional limitation, physical pain, psychological discomfort, physical disability, psychological disability, social disability, and handicap. Responses were recorded on a 5-point Likert scale ranging from “never (0)” to “very often (4),” with higher scores indicating greater perceived impact. In this study, the Brazilian Portuguese validated version of the OHIP-14 was used.^26^^,^^27^ Although this version has not been specifically validated for the Cape Verdean population, it was selected because Portuguese is the official language of Cabo Verde and because no validated Cape Verdean Creole or Cabo Verde-specific Portuguese version was available. The Brazilian Portuguese version was considered the most appropriate available validated version by the research team in consultation with Portuguese and Creole speaking collaborators from Sal. Nevertheless, the absence of formal validation in the Sal population should be considered a limitation when interpreting the OHRQoL findings.All collected data were recorded on standardized clinical forms and subsequently entered into a secure digital database for analysis, and the entered data was checked by a second person.

### Statistical analyses

Descriptive analyses were performed to characterize the study population. Continuous variables (dmft/DMFT, pufa/PUFA, OHIP-14) were summarized using means, standard deviations (SD), and ranges, reported separately for children (1-5 y/o; 6-11 y/o; 12-17 y/o) and adults. Caries prevalence, pufa/PUFA prevalence and RCI were reported as percentages with 95% confidence intervals (CI). Categorical variables (sex, oral hygiene behaviors, access to care, insurance status, tobacco use in adults) were summarized as frequencies and percentages.

Associations between oral health related risk factors and caries experience were examined using multiple linear regression (children and adults separately), with dmft/DMFT as the dependent variable. All independent variables (possible risk factors) were entered simultaneously (enter method), and regression coefficients (B) with 95% CI were reported. Binary logistic regression assessed associations with untreated caries consequences (pufa/PUFA: yes/no) in children and adults, and with periodontitis (yes/no) in adults, using the same independent variables. Odds ratios (OR) and 95% CI were calculated. Cases with missing data were excluded listwise.

Among adults only, OHRQoL was assessed using OHIP-14 total scores (continuous). Normality was evaluated visually and with the Kolmogorov–Smirnov test. Due to non-normal distribution, non-parametric tests were applied: Spearman’s correlation (OHIP-14 vs. DMFT), Mann–Whitney U (pufa/PUFA), and Kruskal–Wallis H (DPSI categories). Analyses were performed in SPSS v29.0.2.0 with α = 0.05 (2-tailed).

## Results

A total of 1,371 dentulous participants were included in the study, comprising 609 children (aged 1-17 years; 45 aged 1-5 years, 404 aged 6-11 years and 160 aged 12-17 year; 46.9% female, sex data missing for 2 participants) and 762 adults (aged 18-89 years, 51.6% female). The mean age of the children was 9.54 ± 3.3 years and 38.0 ± 14.6 years for adults. The mean age of the different age groups of the children (1-5 y/o (48.9% female); 6-11 y/o (48.8% female); 12-17 y/o (41.3% female)) was respectively 4.43 ± 0.9 years; 8.3 ± 1.6 years; 14.1 ± 1.6 years. The youngest age group, children aged 1-5 years, was relatively small (n=45). Estimates for this subgroup should therefore be interpreted with caution and are presented descriptively. Since recruitment took place through open invitations and via multiple public channels rather than via a predefined sampling frame, it was not possible to calculate a response rate.

### Oral health status

An overview of the descriptive results is provided in Table 1. The mean dmft/DMFT score was 3.7 ± 3.2 among children (range: 0-24; per age group respectively 3.8 ± 4.6; 3.7 ± 2.9 and 3.8 ± 3.4) and 7.4 ± 6.0 (range 0-28) among adults. The caries prevalence (dmft/DMFT>0) for this population was 87.5%. The mean pufa/PUFA score was 1.0 ± 1.5 in children (range 0-18 per age group respectively 0.6 ± 1.3; 1.0 ± 1.3; 1.2 ± 1.9) and 1.6 ± 2.5 (range 0-20) in adults. The PUFA prevalence (pufa/PUFA>0) was 52.2% (prevalence rates of caries experience, untreated caries consequences and restorative care index by age group are provided in Table 1). In this population, 81.0% of participants had untreated dental caries (dt/DT > 0), 81.4% in children (resp. 66.7%; 82.4%; 83.1%) and 80.6% in adults. Regarding periodontal health, assessed using the DPSI (n = 757), all adults had a DPSI score of at least 1 in at least one of the sextants. In total, 62.7% of the adults had a highest score of DPSI 3+ or 4 in at least one sextant.

**Table 1:** Descriptive statistics concerning dental caries, consequences of untreated caries and oral health related quality of life.

	Total (n = 1371)	1-5 y/o (n = 45)⁎⁎	6-11 y/o (n = 404)	12-17 y/o (n = 160)	Adults (n = 762)
	**mean ± SD**				
dmft/DMFT	5.8 ± 5.5	3.8 ± 4.6	3.7 ± 2.9	3.8 ± 3.4	7.4 ± 6.0
dt/DT	3.5 ± 3.5	3.6 ± 4.5	3.4 ± 2.8	3.4 ± 3.1	3.7 ± 3.8
mt/MT	2.0 ± 4.0	0.2 ± 0.5	0.2 ± 0.7	0.3 ± 0.7	3.5 ± 4.6
ft/FT	0.2 ± 1.0	0.1 ± 0.3	0.1 ± 0.6	0.1 ± 0.5	0.3 ± 1.2
pufa/PUFA	1.35 ± 2.1	0.62 ± 1.3	0.96 ± 1.3	1.2 ± 1.9	1.6 ± 2.5
	**% [95% CI]**				
Caries prevalence⁎	87.5 [85.8-89.4]	66.7 [53.3- 80.0]	84.4 [80.7- 87.9]	83.8[78.8- 88.8]	91.2 [89.1-93.2]
pufa/PUFA prevalence	52.2 [49.6- 54.8]	24.4 [11.-37.8]	47.5 [42.8-52.5]	56.9[49.4- 64.4]	55.2 [51.7-58.7]
RCI#	6.4 [5.2-7.7]	2.0 [0.0-5.3]	2.2 [1.1-3.7]	4.7[2.1-8.0]	9.1 [7.2-11.1]

⁎ Caries prevalence: the proportion of individuals who have experienced dental caries (% DMFT>0).

# RCI: restorative care index: the proportion of decayed teeth that have been filled: (FT/(DT+FT)).

⁎⁎ Estimates for the 1-5-year age group should be interpreted with caution because of the small sample size.

### Factors related with oral health

Most participants reported regular toothbrushing, with a median frequency of 14 times/week for children and 15 times/week for adults. Ownership of a personal toothbrush was reported by 96.4% of children (n = 605) and 97.6% of adults (n = 762). Availability of toothpaste in the household was reported by 88.3% of children and 86.0% pf adults (n = 600 and n = 757, respectively). Consistent toothpaste use was reported by 87.6% of children and 84.4% of adults, while 6.1% vs 6.2% used it sometimes, and 6.3% vs 9.4% never (n = 591 vs n = 745).

Regarding professional oral health care, 35.5% of children and 67.5% of adults (n = 606 and n = 759) had visited a dentist at least once in their lifetime, with a median time since the last visit of 1 year for children and 3 years for adults. General health insurance was reported by 58.2% of children and 38.9% of adults (n = 600 and n = 759), while 62.7% and 46.2% respectively, had dental insurance. Among adults, 14.5 % (n = 110/760) reported current tobacco use. At the time of the research visit, 28.8% of children and 31.3% of adults reported experiencing pain in the oral cavity.

Table 2 shows the results of the linear regression to assess possible associations between oral health-related risk factors and dmft/DMFT scores. The models included the 5 variables: brushing frequency, ownership of a toothbrush, availability of toothpaste, history of dental visits, and dental insurance status as independent variables, and for adults, smoking was also added. Among children, dental insurance was significantly associated with lower dmft/DMFT scores (B = -0.67, 95% CI: -1.23 – -0.11, *p* = .019). Among adults, toothbrush ownership was associated with lower DMFT scores (B = -4.64, 95% CI: -7.69 to -1.60, *p* = .003), while having ever visited a dentist was associated with higher DMFT scores (B = 1.33, 95% CI: 0.36-2.30, *p* = .008).

**Table 2:** Regression analyses of possible risk factors related to oral health.

		dmft/DMFT⁎					pufa/PUFA#			Periodontitis (yes/no)		
		B	SE	95% CI	Beta	*p*-value	OR	95% CI	*p*-value	OR	95% CI	*p*-value
Children (n = 609)	(Constant)	4.12	0.83	2.49 – 5.74	−	−	0.23	−	−	−	−	−
	Brushing frequency per week (n = 594)	−0.02	0.02	−0.06 – 0.02	−0.04	.38	1.01	0.98 – 1.04	.48	−	−	−
	Own toothbrush (yes = 1; n = 605)	0.02	0.91	−1.78 – 1.81	0.001	.99	3.40	1.02 – 11.32	**.046**	−	−	−
	Toothpaste available (yes = 1; n = 600)	0.20	0.51	−0.80 – 1.19	0.018	.70	0.58	0.30 – 1.09	.09	−	−	−
	Ever visited a dentist (yes = 1; n = 606)	0.43	0.29	−0.13 – 0.99	0.064	.13	1.24	0.87 – 1.76	.23	−	−	−
	Dental insurance (yes = 1; n = 585)	−0.67	0.28	−1.23 – -0.11	−0.100	**.019**	0.61	0.43 – 0.86	**.005**	−	−	−
Adults (n = 762)	(Constant)	10.70	1.52	7.73 – 13.68	−	−	3.09	−	−	2.76	−	−
	Brushing frequency per week (n = 760)	−0.02	0.04	−0.09 – 0.06	−0.015	.69	0.99	0.97 – 1.01	.32	0.98	0.95 – 1.00	.08
	Own toothbrush (yes = 1; n = 762)	−4.64	1.55	−7.69 – -1.60	−0.115	**.003**	0.72	0.24 – 2.17	.56	0.75	0.22 – 2.51	.64
	Toothpaste available (yes = 1; n = 757)	0.50	0.70	−0.87 – 1.87	0.027	.48	0.67	0.42 – 1.06	.09	1.20	0.75 – 1.90	.45
	Ever visited a dentist (yes = 1; n = 759)	1.33	0.50	0.36 – 2.30	0.100	**.008**	0.98	0.71 – 1.34	.88	1.00	0.72 – 1.40	.98
	Dental insurance (yes = 1; n = 747)	0.53	0.46	−0.38 – 1.44	0.043	.25	0.83	0.62 – 1.12	.23	0.83	0.61 – 1.13	.24
	Current smoking (yes = 1; n = 760)	−0.29	0.65	−1.57 – 0.99	−0.016	.66	1.49	0.97 – 2.29	.07	1.66	1.05 – 2.63	**.03**

Significant *p*-values are depicted in bold.

⁎ Lineair regressions.

# Logistic regressions, n refers to the number of individuals included in each group.

Table 2 also presents the results of the logistic regression to assess possible associations with the pufa/PUFA scores using the same variables. Among children, toothbrush ownership was associated with higher odds of PUFA presence (OR = 3.40, 95% CI: 1.02 – 11.32, *p* = .046), while dental insurance was associated with lower odds of PUFA presence (OR = 0.61, 95% CI: 0.43-0.86, *p* = .005). No significant associations were found among adults.

In addition, Table 2 displays the results of the logistic regression used to assess possible associations with the presence or absence of periodontitis using the same variables, with only the variable ‘current smoking’ significantly associated with higher odds of having periodontitis (OR = 1.66, 95% CI: 1.05-2.63, *p* = .03).

### OHRQoL

The mean OHIP-14 score in adults was 14.6 ± 12.6 (range 0-56, n = 491). Visual inspection of the histogram, as well as the results of the Kolmogorov–Smirnov test (D = 0.123, *p* < .001), indicated that the distribution of the summed OHIP-14 score deviated significantly from normality. A weak but significant positive correlation between OHIP-14 and DMFT (ρ = 0.173, *p* < .001) shows that higher DMFT scores were associated with slightly poorer OHRQoL. Participants with PUFA lesions had significantly higher OHIP-14 scores (U = 22,229.5, *p* < .001; Median = 14.0 vs. 9.0). A Kruskal–Wallis test indicated significant differences in OHIP-14 scores across DPSI categories (H(4) = 14.07, *p* = .007). Median OHIP-14 scores differed across maximum DPSI categories, with scores of 4.0 for DPSI 1, 10.0 for DPSI 2, 8.0 for DPSI 3-, 12.0 for DPSI 3+, and 12.5 for DPSI 4. These findings indicate a small progressive decline in OHRQoL with increasing periodontal disease severity.

## Discussion

This population-based study provides the first comprehensive assessment of oral health and related risk factors among dentulous residents of Sal, Cape Verde. Oral disease burden was high across all ages, with almost 9 out of 10 participants affected by dental caries and more than half of the participants showing signs of untreated caries consequences. The discrepancy between high self-reported toothbrushing rates and the substantial burden of untreated disease suggests possible reporting bias. In addition, reported ownership of a toothbrush does not guarantee daily use by all family members. These behaviors may also be insufficient to counteract other factors such as dietary habits/high sugar intake, suboptimal brushing techniques, and limited exposure to fluoride. Dental insurance coverage among children was associated with lower dmft/DMFT scores, highlighting the potential role of access to care in reducing inequalities. In adults, prior dental visits were associated with higher DMFT, likely reflecting treatment-seeking for existing disease because of pain. Current smoking was the only factor significantly associated with periodontitis. Adults with more caries and severe periodontal disease reported worse oral health-related quality of life (OHRQoL). Overall, these results underscore the urgent need for targeted preventive strategies and improved oral health infrastructure for both children and adults.

### Oral health status

The oral health status of residents in Sal, Cape Verde, reveals a substantial burden of dental caries: the caries prevalence among both children (87.5%) and adults (91.2%) exceeds the estimate for lower-middle income countries of approximately 42.9% for untreated caries in deciduous teeth and approximately 28.9% in permanent teeth (and resp. 38.6% and 28.5% in the African region).^28^^,^^13^ The high PUFA prevalence further indicates widespread untreated caries, although published pufa/PUFA index data from lower-middle-income countries and/or countries in Africa are not available, so comparisons are not possible. The low Restorative Care Index (6.4%) highlights limited availability of curative dental services.

Periodontal health is also concerning, with over 60% of adults showing DPSI maximum scores of 3+ or 4, indicating substantial periodontal treatment or assessment needs. However, these findings should be interpreted in light of the screening nature of the DPSI. The DPSI was developed as a clinical screening tool to guide treatment planning in dental practice. In this study, the highest DPSI score in any sextant was used as the participant-level score; therefore, one localized deepened pocket was sufficient to classify a participant as having periodontitis. This approach is sensitive for identifying individuals requiring further periodontal assessment, but it may overestimate the prevalence of generalized periodontal disease in a population-based study. More detailed periodontal surveillance, including information on the extent and distribution of periodontal pockets and tooth loss, would be needed to better characterize the periodontal disease burden on Sal. According to the Global Burden of Disease study, Cabo Verde ranks among the top 3 countries globally for age-standardized prevalence of periodontal disease, exceeding both global and regional averages. This suggests a disproportionately high burden relative to the country’s development level. According to the Global Burden of Disease study, Cabo Verde ranks among the top 3 countries worldwide with the highest age-standardized prevalence rate (ASPR) of periodontal disease, estimated at 34,122.9 per 100,000 persons.^29^ This exceeds the global ASPR of 17,011.6 and the average for low-middle SDI countries (20,920.5 per 100,000). All these findings align with global trends showing that socioeconomically disadvantaged populations bear a disproportionate burden of oral disease.

### Factors related to oral health

Although dental care is officially free for children in the Cape Verdian Islands, only 35.5% had ever visited a dentist. Despite universal coverage through the public dental care system, some parents or caretakers did not indicate that their children were insured, indicating limited awareness of available benefits. The finding that children who indicated having dental insurance had significantly lower dmft/DMFT scores, suggests that insurance awareness facilitates the access to preventive care. However, socioeconomic confounders could also play a role. Inhabitants who are able to afford additional dental insurance, might also be living in better socioeconomic circumstances, which has been associated with better oral health outcomes.^30^

While school-based oral health programs and preventive interventions have been associated with improved caries outcomes in children on other continents, the impact of insurance coverage on oral health remains unclear.^31^ In low-income adults in the United States, actual coverage of dental costs can lead to improved oral health outcomes, although the specific mechanisms and long-term effects remain an area of ongoing research.^32^ Other (unknown) barriers may also limit the use of services.

In adults, toothbrush ownership was associated with lower DMFT scores, which is consistent with previous research results.^33^ Interestingly, a history of dental visits among adults was associated with higher DMFT scores, likely reflecting reverse causality: individuals with more severe dental problems are more likely to seek care. The lack of other significant associations for example, between brushing frequency and oral health outcomes in both children and adults may be due to self-reporting bias. Additionally, the presence of a toothbrush in a household does not necessarily mean that all family members use this brush consistently or correctly, which may further contribute to the discrepancy between reported behaviors and the observed high burden of disease. Oral health and access to dental care are determined by a complex interplay of behavioral, socioeconomic, and societal factors, which may explain the relatively small number of variables found to be significantly associated with oral health outcomes in this study.

### OHRQoL

Adults in Sal reported a moderate impact of oral health on quality of life, with a mean OHIP-14 score of 14.6 which is higher than typical in high-income countries, and this score provides a useful baseline for future interventions.^34^ Adults with PUFA lesions had significantly worse OHRQoL, consistent with findings in children and older adults.^35^^,^^36^ While this correlation has been described anecdotally in adults, a systematic review is lacking.

### Methodological reflection

This study’s strengths include a large, diverse sample and validated measures, and its unique contribution as the first study to examine oral health in Cape Verde. Although combining clinical assessments with self-reported experiences of OHRQoL is a strength of this study, self-reported behavioral measures such as brushing frequency may be prone to reporting bias. The use of Portuguese questionnaires and the Brazilian Portuguese OHIP-14 version is a limitation as well. Although Portuguese is the official language of Cabo Verde and items could be clarified when needed, Cape Verdean Creole is the main language used in daily life. The questionnaires were not formally validated or pilot-tested in the Sal population, which may have affected comprehension and comparability of self-reported outcomes. The use of DPSI as a population-level periodontal outcome is a further limitation. While useful for screening and identifying periodontal care needs, the highest sextant score does not provide detailed information on disease extent, severity or distribution.

While the cross-sectional design limits causal inference, the identification of oral health-related factors remains valuable for mapping potential targets for future preventive strategies. Community-based recruitment may have led to selection bias, may have introduced selection bias and also observer bias cannot be ruled out. The exclusion of edentulous individuals could also have a profound impact on the outcomes: in a population with a high burden of untreated caries and tooth loss, edentulous adults (particularly older adults) may represent an important and vulnerable subgroup. Their exclusion in our research may have led to an underestimation of the burden of oral disease. Moreover, the single island, sample limits the generalizability to other parts of Cape Verde. Future studies should include multi-island samples to enhance external validity.

### Public health implications

These results are critical for improving oral health in Cape Verde. In line with WHO recommendations to integrate oral health into primary care, context-specific data are essential for designing culturally appropriate interventions.^37^^,^^38^ Despite high reported brushing rates, the widespread disease burden suggests that oral hygiene practices may be insufficient, and other factors may also contribute, such as limited fluoride exposure or high sugar availability. Inconsistent toothpaste availability further highlights structural barriers in access to basic hygiene products. Future targeted health promotion should emphasize prevention, early intervention, and equitable access to essential resources, such as fluoride, silver diamine fluoride (SDF), and atraumatic restorative treatment (ART), particularly for children. Co-designed interventions with local leaders via a mentorship programme has been suggested to aid in health system changes.^39^ Achieving universal health coverage should ensure that everyone can access high-quality health services when and where they need them, without facing economic barriers.^40^ Upstream policy measures aimed at reducing the availability and marketing of foods and beverages with a high sugar content, and improving the overall nutritional environment, will also be essential components of a comprehensive oral health strategy.

## Conclusion

This study provides the first population-based evidence on oral health in Cape Verde, revealing a substantial burden of disease among Sal residents despite high self-reported toothbrushing rates and free dental care for children. Persistent caries and untreated infections highlights gaps in the effectiveness and accessibility of oral health services and hygiene practices. These findings call for targeted, context-specific interventions that combine preventive strategies such as adequate fluoride exposure, improving access to basic oral care, and strengthening early education. In addition, efforts to reduce the availability and consumption of foods with a high sugar content can help create a healthier environment and support long-term improvements in oral health. The data provide a baseline to guide future public health efforts on the island.

## Author contributions

*Catherine M.C. Volgenant:* Conceptualization, Methodology, Analysis, Writing - Original Draft. *Hans J.J. de Soet:* Conceptualization, Methodology, Writing - Review and Editing, Supervision. *Trynke Hoekstra:* Methodology, Writing - Review and Editing, Supervision. *Denise Duijster:* Conceptualization, Methodology, Writing - Review and Editing, Supervision.

## Conflict of interest

None disclosed.
